# Expression of Emotion in Eastern and Western Music Mirrors Vocalization

**DOI:** 10.1371/journal.pone.0031942

**Published:** 2012-03-14

**Authors:** Daniel Liu Bowling, Janani Sundararajan, Shui'er Han, Dale Purves

**Affiliations:** 1 Neuroscience and Behavioral Disorders Program, Duke-National University of Singapore, Graduate Medical School Singapore, Singapore, Singapore; 2 Department of Neurobiology, Research Drive, Duke University Medical Center, Durham, North Carolina, United States of America; 3 Center for Cognitive Neuroscience, Levine Science Research Center, Duke University, Durham, North Carolina, United States of America; Indiana University, United States of America

## Abstract

In Western music, the major mode is typically used to convey excited, happy, bright or martial emotions, whereas the minor mode typically conveys subdued, sad or dark emotions. Recent studies indicate that the differences between these modes parallel differences between the prosodic and spectral characteristics of voiced speech sounds uttered in corresponding emotional states. Here we ask whether tonality and emotion are similarly linked in an Eastern musical tradition. The results show that the tonal relationships used to express positive/excited and negative/subdued emotions in classical South Indian music are much the same as those used in Western music. Moreover, tonal variations in the prosody of English and Tamil speech uttered in different emotional states are parallel to the tonal trends in music. These results are consistent with the hypothesis that the association between musical tonality and emotion is based on universal vocal characteristics of different affective states.

## Introduction

Different sets of tones and tone-relationships are used to convey particular emotions in a variety of musical cultures [1]–[3]. In Western music, this phenomenon is evident in the association of the major mode with positive or excited emotion, and the minor mode with negative or subdued emotion [4]–[7]. It is unclear whether the basis for these associations is intrinsic to tonal perception or the result of exposure to Western music. One way to approach this question is to compare how tones and tone-relationships are used to express emotion in different musical traditions. The classical music of South India (called *Carnatic* music) is especially useful for such comparison because, as in Western music, the emotions associated with various tone collections (called *ragas*) are well documented [8]–[11]. Accordingly we asked how the tones in Carnatic melodies composed in *ragas* associated with positive/excited and negative/subdued emotions compare with the use of tones in Western melodies composed in the major and minor modes. In particular, we wished to know whether there is a common denominator in the way musical tonality is used to convey emotion across cultures, and, if so, its probable basis.

The tonality of a Carnatic melody is organized by the *raga* in which it is composed. *Ragas* are analogous to modes in Western music in that they specify a particular collection of tones and tone-relationships. *Ragas*, however, are more complex because they also prescribe specific patterns of ascending and descending movement and *gamakam* (ornamentations in Indian music similar to vibrato, portamento and accent in Western music) [12], [13]. Many *ragas* are associated with specific emotional themes called *rasas* [8]–[11]. Traditional Indian aesthetics defines 9 *rasas*, two of which parallel the emotions associated with the major and minor modes and are thus suited to a cross-cultural comparison. These are *Haasya*, the *rasa* of joy, happiness, and mirth; and *Karunaa*, the *rasa* of sadness, grief, and pity [8]–[11], [14]. The *ragas* selected for comparison are associated with these *rasas*.

**Figure 1:**
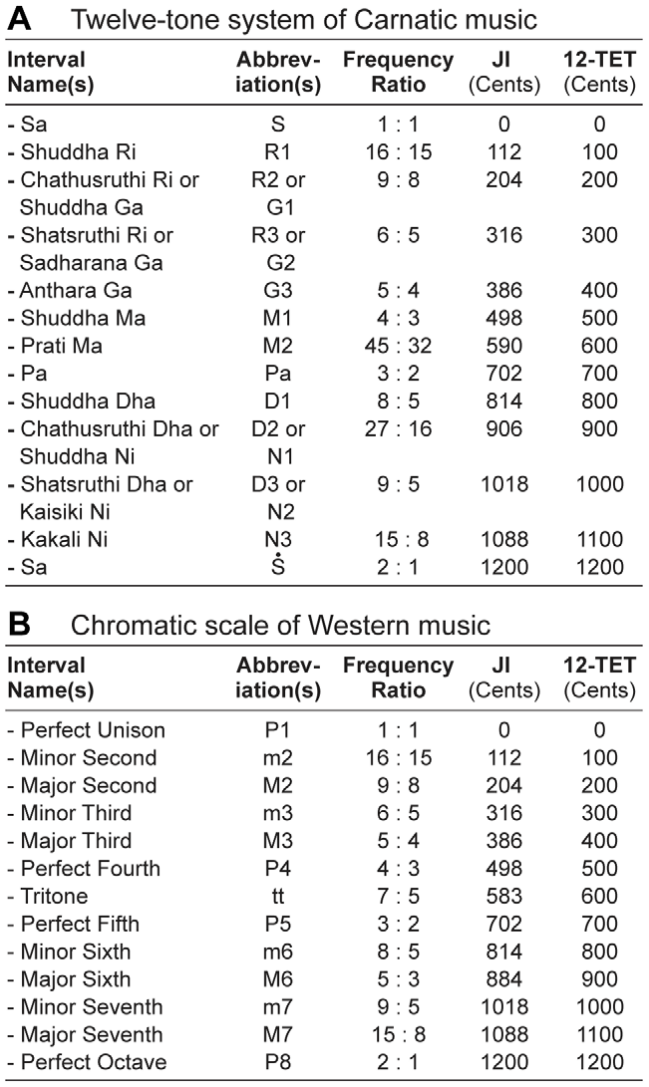
Musical intervals in Carnatic and Western music. (A) The 12 principal intervals of Carnatic music (13 including unison). Each interval is a tone defined by the ratio of its fundamental frequency to the tonic (Sa). Interval names, abbreviations, frequency ratios, and sizes in cents for just intonation (JI) as well as 12-tone equal temperament (12-TET) tunings are shown. When two names are given they refer to enharmonic equivalents. Here and in Figure 2, a dot above or below the abbreviated interval name indicates that it belongs in the octave above or below, respectively. (B) The 12 intervals of the Western chromatic scale, comparably presented.

The basic set of tones and tone-relationships in Carnatic music from which *ragas* are derived is the 12-tone octave division shown in Figure 1A [12]. An additional 10 tones (microtonal variants of 10 tones in the basic set) are used in *gamakams* (see above) [12]. These were not included in the present analysis because the variations they introduce are small relative to the principal tones and tone-relationships in a melody, and because they are not always notated. The chromatic scale, which defines the basic set of tones and tone-relationships in Western music from which the major and minor modes are derived, is shown is Figure 1B for comparison [7], [12].

## Methods

### Music data

**Figure 2:**
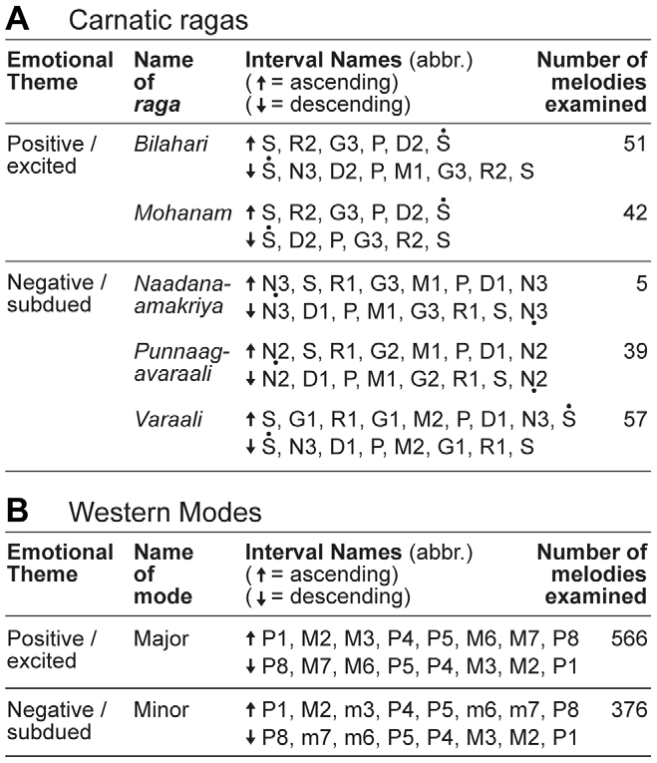
The *ragas* and modes examined. (A) Carnatic *ragas* commonly associated with positive/excited and negative/subdued emotion, and the number of melodies examined in each. The interval name abbreviations are from Figure 1. (B) Western modes commonly associated with positive/excited and negative/subdued emotion are included for comparison (data from [19]).

For the *Haasya rasa*, the selected *ragas* were *Bilahari* and *Mohanam*. For the *Karunaa rasa*, the selected *ragas* were *Naadanaamakriya*, *Punnaagavaraali*, and *Varaali* (Figure 2A) [8], [9], [11]. Several of these *ragas* are also associated with other *rasas*, but in each case a similarly positive/excited or negative/subdued emotional theme is implied. For example, *Bilahari* can also be associated with *Viram* [8], [11], the *rasa* of courage, pride, and confidence; and *Varaali* can also be associated with *Bibhatsam* [8], the *rasa* of disgust, depression, and self-pity. Melodies composed in these *ragas* were collected from Carnatic music study books [15]–[18]. Carnatic compositions are typically divided into three sections [12]. Each section was treated as a separate melody in the database, and comprised between 21 and 568 notes (*M* = 97.7, *SD* = 71.5). All melodies were traditionally notated using the intervals listed in Figure 1A. In total, 194 Carnatic melodies were assembled, 93 associated with positive/excited emotion, and 101 associated with negative/subdued emotion. The characteristics of these melodies were compared to a previous analysis of tonality in classical Western melodies (Figure 2B) [19].

### Music Analysis

The tonal structure of the Carnatic melodies was assessed by the intervals between notes. For each note, interval size was determined in relation to the previous melody note (melodic intervals) and the tonic note of the *raga* (tonic intervals). In both cases interval size was calculated as the difference in cents between the fundamental frequencies (F0s) of the two notes in question. Melodic intervals thus describe variations in F0 over time, whereas tonic intervals describe the place of each F0 within the tonal context of the *raga*. Interval size data was tallied in 100-cent bins from −1200 to 1200 for melodic intervals, and from 0 to 1200 for tonic intervals. Bin counts were normalized as percentages of the total number of melodic or tonic intervals in a melody and averaged across melodies in one of the emotional conditions examined to obtain average distributions of interval size. Differences between emotional conditions were evaluated for statistical significance using Mann-Whitney *U*-tests. For melodic intervals comparisons were between the proportion of intervals smaller and larger than a major second (chosen as a cutoff because it separates the largest differences exhibited by positive/excited and negative/subdued melodies; see Figure 4). For tonic intervals, comparisons were between proportions of intervals in each 100-cent bin.

### Speech data

Speech was recorded from 20 native speakers of Indian Tamil (10 male) and 20 native speakers of American English (10 male); the speakers ranged in age from 19–72 and had no significant speech or hearing pathology. To mitigate the possible influence of foreign intonation patterns, speakers were required to have lived in the country of their native language for at least 10 years. All subjects gave written informed consent as required by the Institutional Review Boards of Duke University and the National University of Singapore. Speakers read monologues and bi-syllabic expressions in their native language. The monologues consisted of 10 paragraphs, 5 with emotionally positive/excited content and 5 with negative/subdued content (see [19] for examples). The bi-syllabic expressions consisted of 20 emotionally neutral expressions such as “Okay” and “Let's go” in English or “*Kodu*” and “*Nijam*” in Tamil. In both cases the speakers were instructed to use the quality of their voice to convey either positive/excited or negative/subdued affect. The recordings were made in a sound-attenuating chamber with an omni-directional capacitor microphone coupled to a solid-state digital recorder. Data were saved on flash memory cards in .wav format (sampling rate = 44.1 kHz; bit depth = 16 bit).

The emotion expressed in the recordings was evaluated by a different group of 6 subject raters (3 native Tamil speakers [1 male] and 3 native English speakers [1 male]). Given the large number of recordings (2000), a subset comprising 240 recordings (one monologue and two bi-syllabic expressions for each speaker in each emotional condition) was randomly selected for evaluation. Raters listened to each recording, categorizing it as expressing joy or sadness and scoring the quality on a five point scale (e.g., from not very joyful to very joyful). The accuracy of emotion recognition and average quality of expression were assessed in terms of speaker language and rater language. The intended emotions were accurately recognized and comparable in terms of quality of expression in both languages (Figure S1).

### Speech analysis

**Figure 3:**
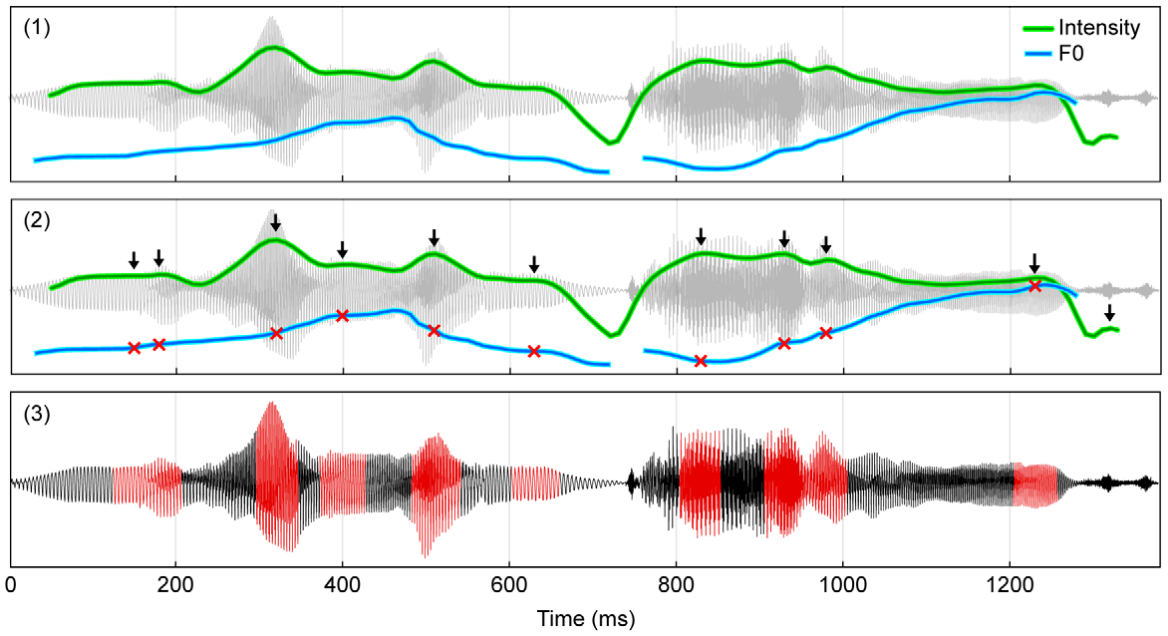
Method of voiced speech extraction. Panel 1 shows the waveform (gray; sound pressure level) of a portion of a speech recording (the phrase “million dollars”) overlaid with indicators of F0 (blue; Hz) and relative intensity (green; dB SPL) calculated at each 10 ms time-step. The missing segments in the F0 contour are segments of “unvoiced” speech (see Text S and S2). Panel 2 shows the same information but with time-points representing local maxima in the intensity contour (arrows) that are also voiced indicated (red crosses). Panel 3 shows the 50 ms windows (red) of voiced speech extracted for spectral analysis; the segments are centered on the voiced intensity maxima in the middle panel.

For each recording, the fundamental frequency and relative intensity of each 10 ms time-step was calculated using Praat's [20] built in “To Pitch” and “To Intensity” algorithms (pitch floor = 60 Hz, pitch ceiling = 600 Hz; time points with autocorrelation values below the default voicing threshold [0.45] were defined as “unvoiced”). These values were used to find time-points with sufficient periodic energy to be classified as voiced that were also local peaks in the intensity contour. These voiced intensity maxima, which correspond mostly to syllable nuclei and voiced consonants, were used to assess speech prosody (see below). Voiced speech segments, defined as 50 ms windows centered on voiced intensity maxima, were extracted for use in the spectral analysis (see below) (Figure 3). The relatively short window size (50 ms) represents a compromise between maximizing frequency resolution and minimizing temporal changes in fundamental frequency.

### Prosodic analysis

For each recording, prosodic intervals were calculated as the frequency difference in cents between adjacent voiced intensity maxima. Like melodic intervals in music, prosodic intervals thus describe changes in frequency over time. Interval size data was tallied in 25-cent bins from −1200 to 1200. Bin counts were normalized as percentages of the total number of prosodic intervals in a recording and averaged across recordings in an emotional condition to obtain representative distributions of interval size. In keeping with the analysis of melodic intervals in music, differences between emotional conditions were evaluated for statistical significance using Mann-Whitney *U*-tests comparing the proportion of intervals smaller and larger than a major second (see above).

### Spectral analysis

For each recording, normalized spectra were calculated for each voiced speech segment. Each segment's spectrum was calculated by applying a Hamming window and a fast Fourier transform (Matlab [21] algorithms ‘hamming.m’ and ‘fft.m’), and normalized in terms of frequency and amplitude. Frequency was normalized by dividing spectral frequency values by the fundamental; amplitude was normalized by dividing spectral amplitude values by the amplitude maximum. Normalized spectra were averaged across recordings in an emotional condition to obtain representative values. Differences between emotional conditions were evaluated for statistical significance by comparing the normalized power at peaks above the tenth harmonic as a percentage of the normalized power at the first 30 harmonic peaks, using Mann-Whitney *U*-tests (the tenth harmonic was chosen as the cutoff because all the intervals between adjacent harmonics after the tenth are smaller than a major second).

## Results

### Music

**Figure 4:**
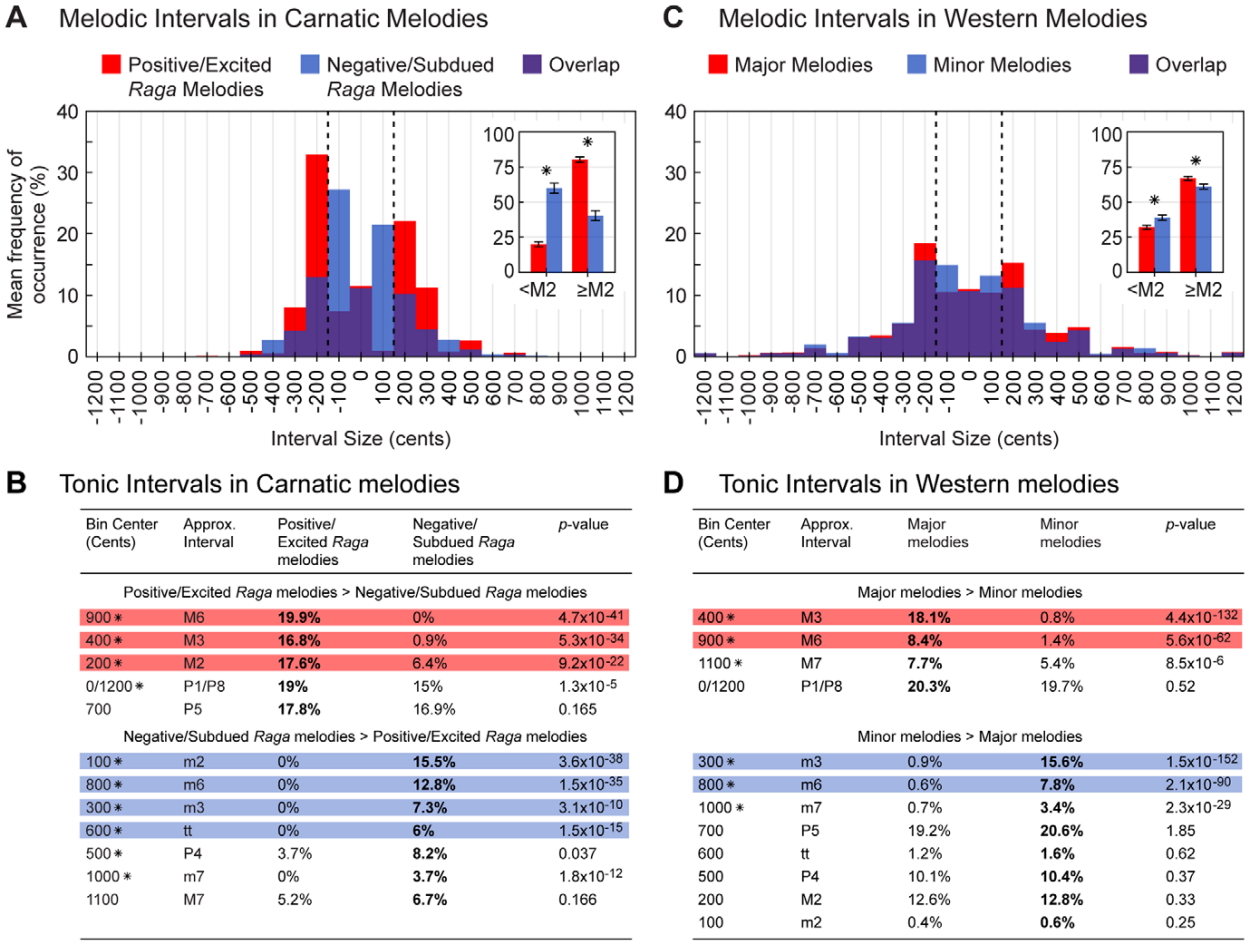
Distributions of musical interval sizes in Carnatic and Western melodies associated with different emotions. (A) Overlay of the average distributions of melodic interval sizes in melodies composed in *ragas* associated with positive/excited (red) and negative/subdued (blue) emotion (purple shows overlap). Inset shows the mean percentages of melodic intervals smaller and larger than a major second (dashed lines separate these groups). Error bars indicate ±2 *SEM*. Asterisks indicate statistically significant differences between the underlying distributions (*P<0.05; Mann-Whitney *U*-tests; see Figure S2 for complete statistics). (B) The mean percentages of different tonic interval sizes in melodies composed in *ragas* associated with positive/excited and negative/subdued emotion. Intervals are grouped according to their prevalence in positive/excited *raga* melodies or negative/subdued *raga* melodies, and listed by size differences in the two emotional conditions. Colored boxes highlight intervals that differ by more than 5% (red indicates greater prevalence in positive/excited melodies, blue indicates greater prevalence in negative/subdued melodies). Asterisks indicate statistically significant differences between the underlying distributions (*P<0.05; Mann-Whitney *U*-tests; see Figure S3 for complete statistics). (C and D) Data from an analysis of major and minor classical Western melodies [19] shown in the same format.

The average distributions of melodic interval sizes in Carnatic melodies composed in *ragas* associated with positive/excited and negative/subdued emotions are shown in Figure 4A. The most obvious difference between emotional conditions concerns the relative prevalence of interval sizes. Positive/excited *raga* melodies comprise more intervals equal to or larger than a major second (200 cents), whereas negative/subdued *raga* melodies comprise more intervals smaller than a major second (see Figure 4A inset). The only exception to this pattern occurs in melodic major thirds, which are slightly more prevalent in negative/subdued *raga* melodies than positive/excited *raga* melodies. In comparison to the overall pattern, however, the discrepancy in melodic major thirds is small. The average prevalence of different tonic interval sizes is shown in Figure 4B. Positive/excited *raga* melodies are characterized by more major intervals, including major seconds, major thirds, and major sixths, whereas negative/subdued *raga* melodies are characterized by more minor intervals, including minor seconds, minor thirds, tritones, and minor sixths. This pattern suggests that the differences in melodic interval sizes between emotional conditions arise from differences in major and minor tonic intervals.

The average distributions of melodic interval sizes in Western melodies composed in the major and minor modes are shown in Figure 4C. Comparison with Figure 4A shows that Carnatic and Western melodies use melodic intervals in much the same way to convey emotion. Melodies composed in tonalities associated with positive/excited emotion (i.e. major melodies) comprise more intervals equal to or larger than a major second, whereas melodies composed in tonalities associated with negative/subdued emotion (i.e. minor melodies) comprise more intervals smaller than a major second (see Figure 4C inset). The average prevalence of different tonic intervals sizes in Western melodies is shown in Figure 4D. Tonic intervals in Western and Carnatic melodies exhibit both similarities and differences. In both traditions major intervals are more prevalent in positive/excited tonalities and minor intervals are more prevalent in negative/subdued tonalities. There are, however, differences in the way these intervals are used. For example, sixths are more important than thirds in making these distinctions in Carnatic music, whereas the reverse is true in Western music (cf. Figure 4B and D). Further, a greater variety of tonic intervals are used to distinguish the emotional character of Carnatic melodies than Western melodies (10 tonic intervals show significant differences in positive/excited and negative/subdued *raga* melodies, whereas only 6 intervals differ significantly in major and minor melodies).

### Speech prosody

**Figure 5:**
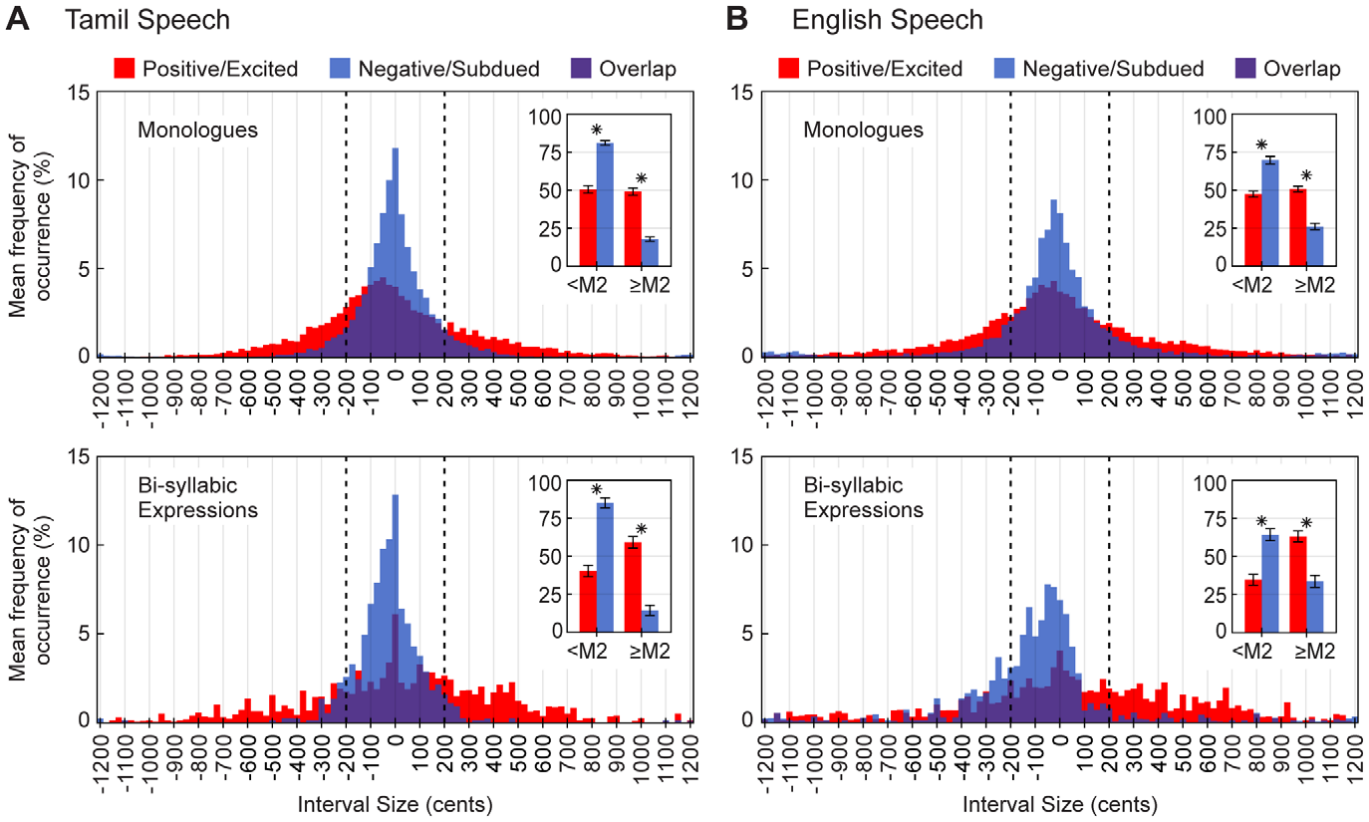
Distributions of prosodic interval sizes in Tamil and English speech expressing different emotions. (A) Overlays of the average distributions of prosodic interval sizes in positive/excited (red) and negative/subdued (blue) Tamil speech (purple shows overlap). The top and bottom panels show data for the monologue and bi-syllabic recordings respectively. Insets compare the mean percentages of prosodic intervals smaller and larger than a major second (dashed lines separate these groups). Error bars indicate ±2 *SEM*. Asterisks indicate statistically significant differences between the underlying distributions (*P<0.05; Mann-Whitney *U*-tests; see Figure S4 for complete statistics). (B) Same as (A), but for English speech.

The average distributions of prosodic interval sizes in positive/excited and negative/subdued Tamil and English speech are shown in Figure 5. In both languages, prosodic intervals in positive/excited speech are larger on average than prosodic intervals in negative/subdued speech. For the monologue recordings, only 49% of the intervals in positive/excited speech were smaller than a major second compared to 75% in negative/subdued speech. Likewise, for the bi-syllabic recordings, only 38% of the intervals in positive/excited speech were smaller than a major second compared to 75% in negative/subdued speech (see Figure 5 insets).

### Speech spectra

**Figure 6:**
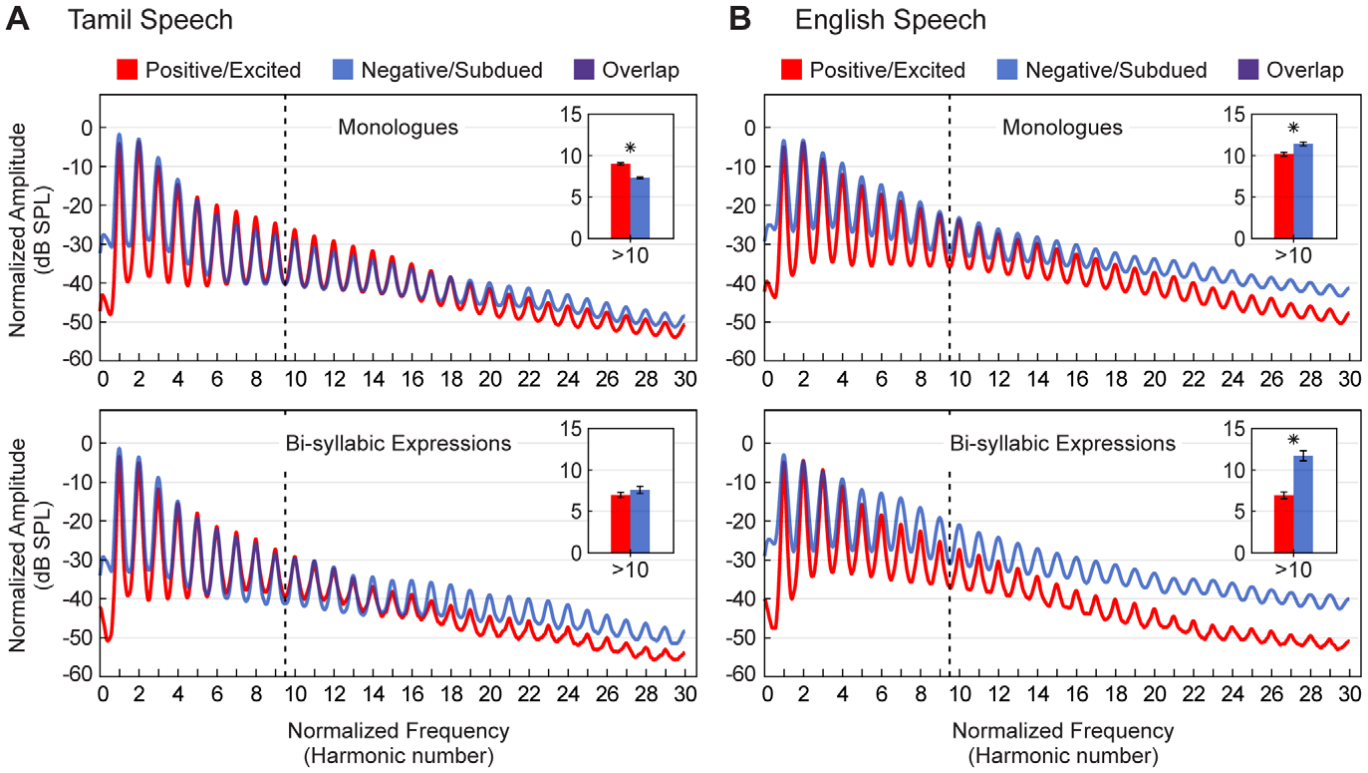
Average normalized spectra in Tamil and English speech expressing different emotions. (A) Overlays of the mean normalized spectra of voiced segments in positive/excited (red) and negative/subdued (blue) Tamil speech (purple shows overlap). The top and bottom panels show monologue and bi-syllabic recordings respectively; dashed lines separate harmonics ten and above. Insets compare the mean power at peaks above the tenth harmonic as a percentage of the mean total power over the first 30 harmonics peaks (computed from spectral amplitudes prior to decibel-conversion). Error bars indicate ±2 *SEM*. Asterisks indicate statistically significant differences between the underlying distributions (*P<0.05; Mann-Whitney *U*-tests; see Figure S5 for complete statistics). (B) Same as (A), but for English speech.

The average normalized spectra of voiced segments in positive/excited and negative/subdued Tamil and English speech are shown in Figure 6. Whereas speech prosody varies in much the same way in Tamil and English speech as a function of emotional state, variations as a function of emotion in speech spectra are less consistent. In English there is relatively more energy in higher harmonics in negative/subdued compared to positive/excited speech in both monologues and bi-syllabic expressions (Figure 6B insets). This pattern tends to emphasize smaller intervals in negative/subdued English speech because the ratios relating higher numbered harmonics are necessarily smaller than those relating lower numbered harmonics (e.g., 16/15 = 1.07, whereas 3/2 = 1.5). The same tendency is apparent in bi-syllabic Tamil speech, but in not the Tamil monologues (cf. Figure 6A and B).

## Discussion

### Cross-cultural patterns in music

This analysis of Carnatic and Western music shows that melodic intervals in both traditions are generally larger in melodies associated with positive/excited emotion, and smaller in melodies associated with negative/subdued emotion. Similarities are also apparent with respect to major and minor tonic intervals, being more prevalent in melodies associated positive/excited and negative/subdued emotions respectively in both traditions. There are however, differences in the particular tonic intervals emphasized most in making these emotional distinctions (see Figure 4B and D). The differences indicate that the use of a particular tonic interval(s) is not critical for the expression of emotion in tonality; what matters is that the set of tonic intervals provide the right opportunities for larger/smaller melodic intervals to occur. Thus, in Western music, emphasis on tonic thirds to differentiate emotions may not reflect an inherent emotional quality of thirds (as sometimes suggested [22], [23], [24]), but other considerations, such as promoting harmony. Taken together, the results suggest that an important cross-cultural aspect of musical tonality in conveying emotion in melody is the relative size of melodic intervals.

### Specific musical intervals in speech prosody

In contrast to at least one other report [22], the present analysis of speech prosody shows that variations in fundamental frequency do not emphasize discrete intervals. Inclusion of more (and more varied) speech samples in addition to examination of interval sizes at higher resolution clearly shows that prosodic interval size varies continuously (see Figure 5). The similarities between speech and music based on prosody thus reflect relative rather than absolute interval size.

### The role of speech spectra

Spectral features differentiate excited and subdued speech less well than prosodic differences. In English, speech spectra show a greater proportion of energy in the higher harmonics of negative/subdued relative to positive/excited speech, thus emphasizing smaller intervals. Tamil speech, however, does not exhibit this pattern so clearly (see Figure 6). Given the differences in formant frequencies in Tamil and English speech (see Table S1 and Text S1), these spectral differences probably arise from different vowel phone usage in the two languages.

In previous work on the affective impact of the major and minor modes we focused on spectra, reporting parallel differences in music and American English speech [19]. As we show here, however, similarities between speech prosody and the musical tone collections associated with emotion are more robust than spectral ones (see Text S2 for further discussion). Thus speech prosody is likely to be more influential in driving associations between speech and musical tonality than spectral information.

### Possible explanations

One way of explaining the common cross-cultural pattern in the use of melodic intervals to convey emotion is mimicry of the universal tonal characteristics of the human voice in different emotional states [19], [25]–[27]. In both Tamil and English speech negative/subdued affect is characterized by relatively small prosodic intervals, whereas positive/excited affect is characterized by relatively large prosodic intervals. Differences in prosodic interval size reflect differences in fundamental frequency variability, an acoustic parameter well known to be elevated in positive/excited vocal expressions and depressed in negative/subdued vocal expressions in a variety of languages [27]. Thus the pattern of interval sizes in speech and music as a function of emotion are similar.

This similarity, however, does not demand a causal relationship between these two domains. It remains possible, for example, that the association between melodic interval size and emotion in music is entirely the product of exposure to arbitrary cultural conventions that just happen to accord with speech. This possibility notwithstanding, such an interpretation is at odds with considerable evidence. In addition to the present results examining tonality, correlations between vocal and musical emotional expression have been documented with respect to tempo, intensity, timbre, and F0 in a variety of languages and musical traditions (reviewed in [27]). It seems unlikely that these correlations are all coincidental.

Accepting that similarities of emotional expression in speech and music reflect something deeper than coincidence, the question remains whether the voice mimics music, or whether music mimics the voice. The latter seems more likely. First, associations between emotion and the voice are physiologically constrained in ways that associations between music and the voice are not. For example, the increased muscular tension associated with high levels of arousal increases the fundamental frequency of the voice, but has no bearing on the octave a guitarist chooses to play a melody in (other than by vocal association). Second, infants spontaneously produce vocalizations with acoustic properties related to their affective status long before the development of musical abilities [28], [29]. Finally, music appears to be uniquely human, whereas affective vocalization is not [30], [31]. These further facts argue that vocal expression of emotion underlies its expression in music.

The use of musical tones to convey emotion is similar in Western and Carnatic Indian melodies. Melodic intervals are smaller in melodies composed in tonalities associated with negative/subdued emotion and larger in melodies composed in tonalities associated with positive/excited emotions. Melodic interval size as a function of emotion in music parallels the variation of interval size with emotion in speech prosody. Although musical interval size is also related to speech spectra, this association is less robust across cultures. These observations, together with physiological, developmental, and phylogenetic evidence, suggest that the affective impact of musical tonality is generated by association with the characteristics of voiced speech in corresponding emotional states.

## Supporting Information

Figure S1 **Assessment of emotion expression in speech recordings.** (A) Accuracy of emotion recognition. Native speakers of Tamil (*N* = 3) and English (*N* = 3) rated a subset of the Tamil and English speech recordings (*N* = 240, 120 in each language, 60 expressing joy and 60 expressing sadness; see Text S and S2) as either “joyful” or “sad”. Data for monologue recordings is shown on the left; data for bi-syllabic expression recordings is on the right. Percentages indicate the average proportions of recordings in each language rated correctly with respect to the intended emotion (standard deviations are shown in brackets). (B) Quality of emotional expression. Subjects also rated the quality of emotional expression on a scale of 1 (not very joyful/sad) to 5 (very joyful/sad). Scores indicate the average rating in each category (standard deviations are shown in brackets).
(TIF)

Figure S2 **Complete statistics for melodic interval comparisons.** (A) Overlays of the distributions underlying the mean percentages shown in the insets of Figure 4A and C (red = positive/excited, blue = negative/subdued. purple shows overlap). Each data point represents the percentage of melodic intervals <M2 or ≥M2 in a single melody. Dashed lines indicate the means of the individual distributions. (B) The results of the two-tailed Mann-Whitney *U*-tests used to assess differences between the distributions in A for statistical significance.
(TIF)

Figure S3 **Complete statistics for tonic interval comparisons.** (A) Overlays of the distributions underlying the mean percentages shown in Figure 4B (red = positive/excited, blue = negative/subdued. purple shows overlap). Each data point represents the percentage of tonic intervals equal to the labeled size in a single melody. Dashed lines indicate the means of the individual distributions. (B) The results of the two-tailed Mann-Whitney *U*-tests used to assess differences between the distributions in A for statistical significance. (C and D) Data presented in the same format for the mean percentages shown in Figure 4D (Western melodies).
(TIF)

Figure S4 **Complete statistics for prosodic interval comparisons.** (A) Overlays of the distributions underlying the mean percentages shown in the insets of the monologue panels of Figure 5 (red = positive/excited, blue = negative/subdued. purple shows overlap). Each data point represents the percentage of prosodic intervals <M2 or ≥M2 in a single recording. Dashed lines indicate the means of the individual distributions. (B) The results of the two-tailed Mann-Whitney *U*-tests used to assess differences between the distributions in A for statistical significance. (C and D) Data presented in the same format for bi-syllabic-expressions.
(TIF)

Figure S5 **Complete statistics for average normalized spectra comparisons.** (A) Overlays of the distributions underlying the mean percentages shown in the insets of Figure 6 (red = positive/excited, blue = negative/subdued. purple shows overlap). Each data point represents the average power at peaks above the tenth harmonic as a percentage of the average total power over the first 30 harmonics peaks in the voiced segments from a single recording. Dashed lines indicate the means of the individual distributions. (B) The results of the two-tailed Mann-Whitney *U*-tests used to assess differences between the distributions in A for statistical significance.
(TIF)

Table S1 **Comparison of fundamental and formant frequencies in Tamil and English speech.** (A) Comparison of mean fundamental frequencies and mean peak frequencies for the first and second formants in Tamil and English speech (from the monologue recordings). The results of two-tailed independent samples t-tests used to assess language differences for statistical significance are also shown. (B) Same as (A), but for the bi-syllabic expression recordings. Praat's [20] “To Formant” linear predictive coding algorithm was used with default settings (‘number of formants’ = 5, ‘maximum formant’ for male/female = 5 kHz/5.5 kHz) to calculate the formant frequencies.
(DOC)

Text S1 **Discussion of fundamental and formant frequencies in English and Tamil speech.** This file discusses the results of the comparison of fundamental and formant frequencies in Tamil and English shown in Table S1.
(DOC)

Text S2 **Further discussion of spectral similarities** This file compares past [19] and present analyses of speech spectra and considers questions of perceptual relevance. The potential importance of speech spectra to the affective impact of harmonic rather than melodic arrangements of musical tones is also discussed.
(DOC)
