# Supplementary figures and images for: Expression of Emotion in Eastern and Western Music Mirrors Vocalization
Source: PLoS One. 2012 Mar 14;7(3):e31942. doi: 10.1371/journal.pone.0031942 (PMC3303771; doi:10.1371/journal.pone.0031942)

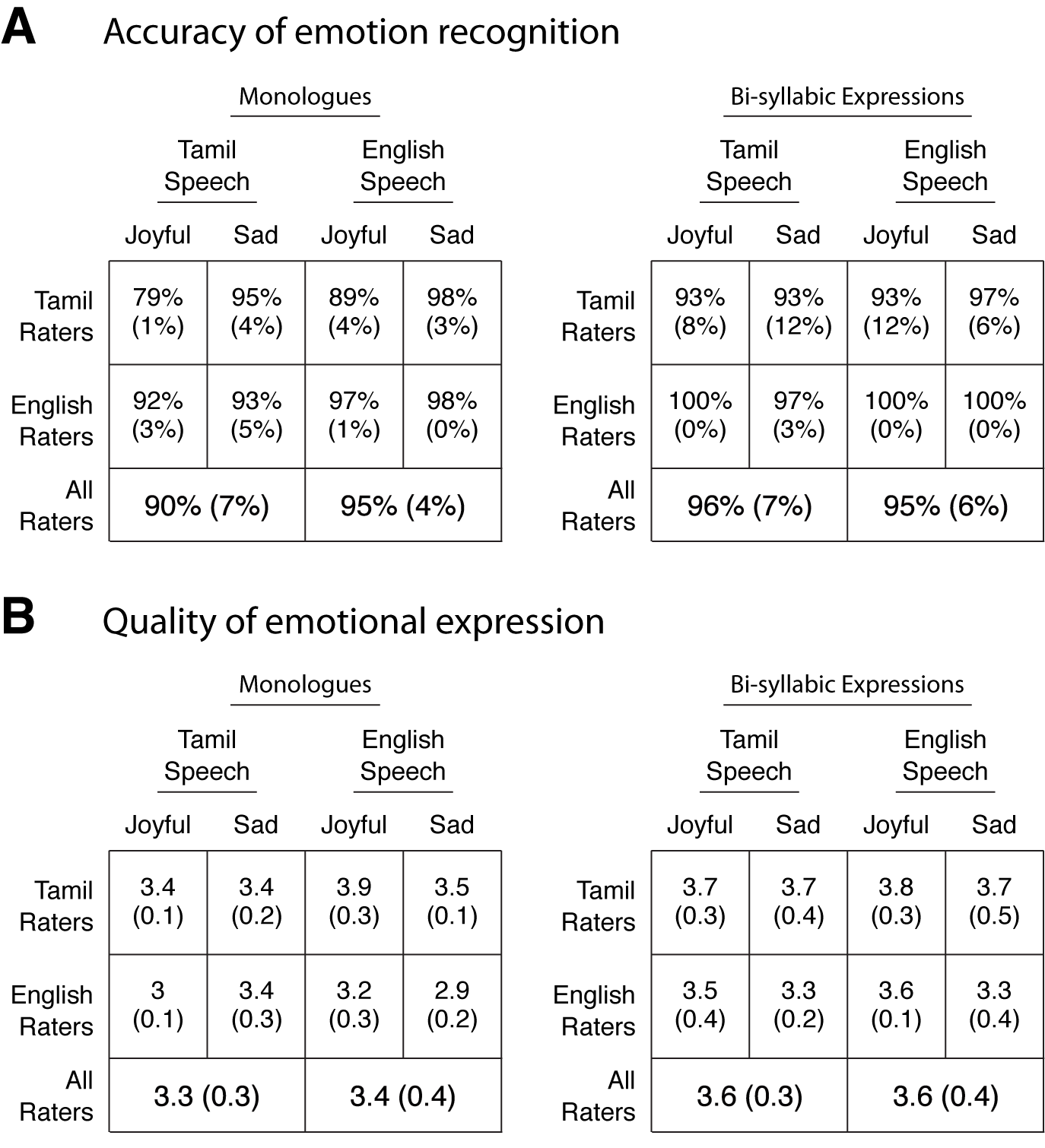

Supplement: Figure S1 — Assessment of emotion expression in speech recordings. (A) Accuracy of emotion recognition. Native speakers of Tamil (N = 3) and English (N = 3) rated a subset of the Tamil and English speech recordings (N = 240, 120 in each language, 60 expressing joy and 60 expressing sadness; see Text S and S2) as either “joyful” or “sad”. Data for monologue recordings is shown on the left; data for bi-syllabic expression recordings is on the right. Percentages indicate the average proportions of recordings in each language rated correctly with respect to the intended emotion (standard deviations are shown in brackets). (B) Quality of emotional expression. Subjects also rated the quality of emotional expression on a scale of 1 (not very joyful/sad) to 5 (very joyful/sad). Scores indicate the average rating in each category (standard deviations are shown in brackets). (TIF) [file pone.0031942.s001.tif]

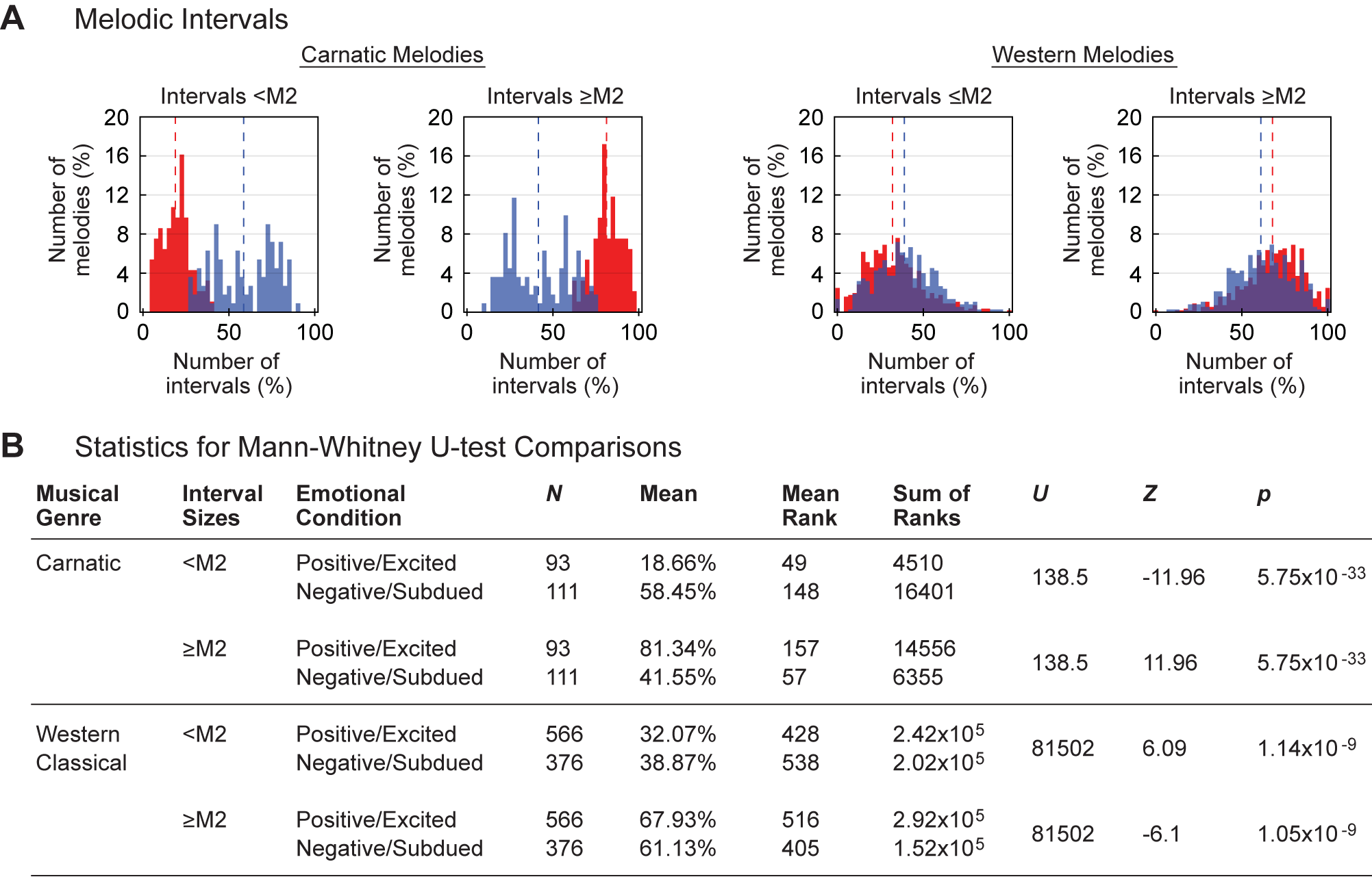

Supplement: Figure S2 — Complete statistics for melodic interval comparisons. (A) Overlays of the distributions underlying the mean percentages shown in the insets of Figure 4A and C (red = positive/excited, blue = negative/subdued. purple shows overlap). Each data point represents the percentage of melodic intervals <M2 or ≥M2 in a single melody. Dashed lines indicate the means of the individual distributions. (B) The results of the two-tailed Mann-Whitney U-tests used to assess differences between the distributions in A for statistical significance. (TIF) [file pone.0031942.s002.tif]

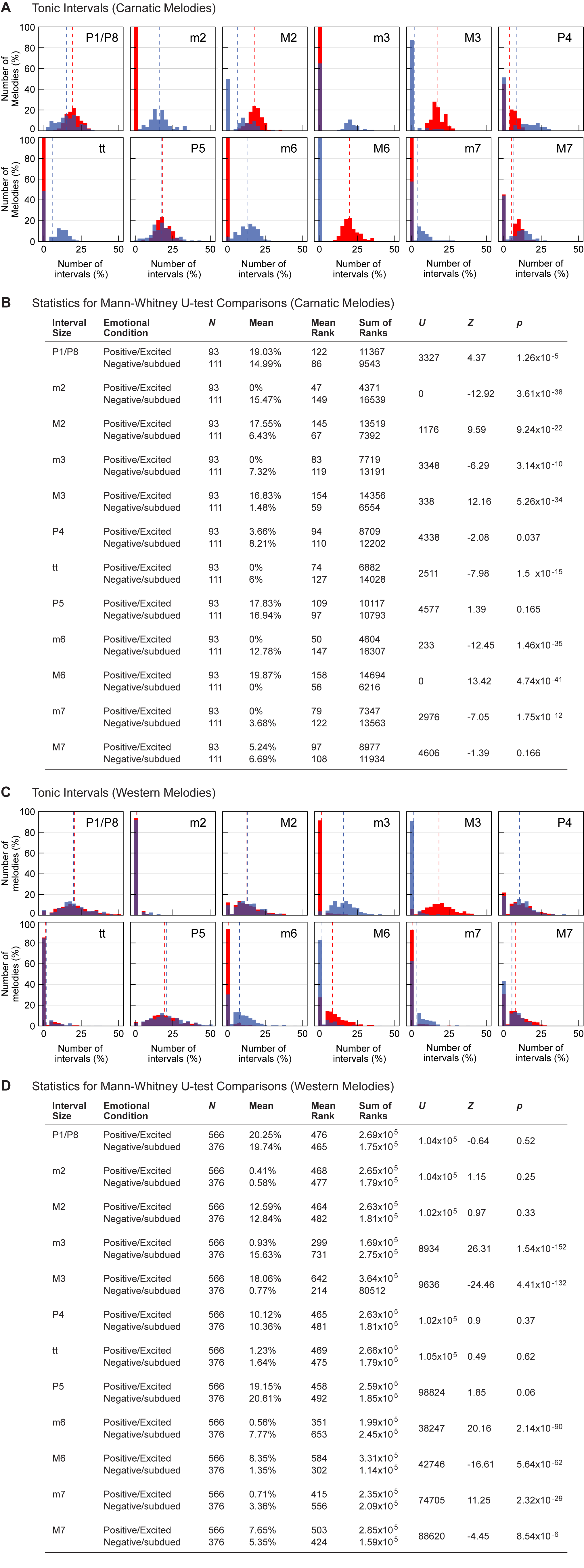

Supplement: Figure S3 — Complete statistics for tonic interval comparisons. (A) Overlays of the distributions underlying the mean percentages shown in Figure 4B (red = positive/excited, blue = negative/subdued. purple shows overlap). Each data point represents the percentage of tonic intervals equal to the labeled size in a single melody. Dashed lines indicate the means of the individual distributions. (B) The results of the two-tailed Mann-Whitney U-tests used to assess differences between the distributions in A for statistical significance. (C and D) Data presented in the same format for the mean percentages shown in Figure 4D (Western melodies). (TIF) [file pone.0031942.s003.tif]

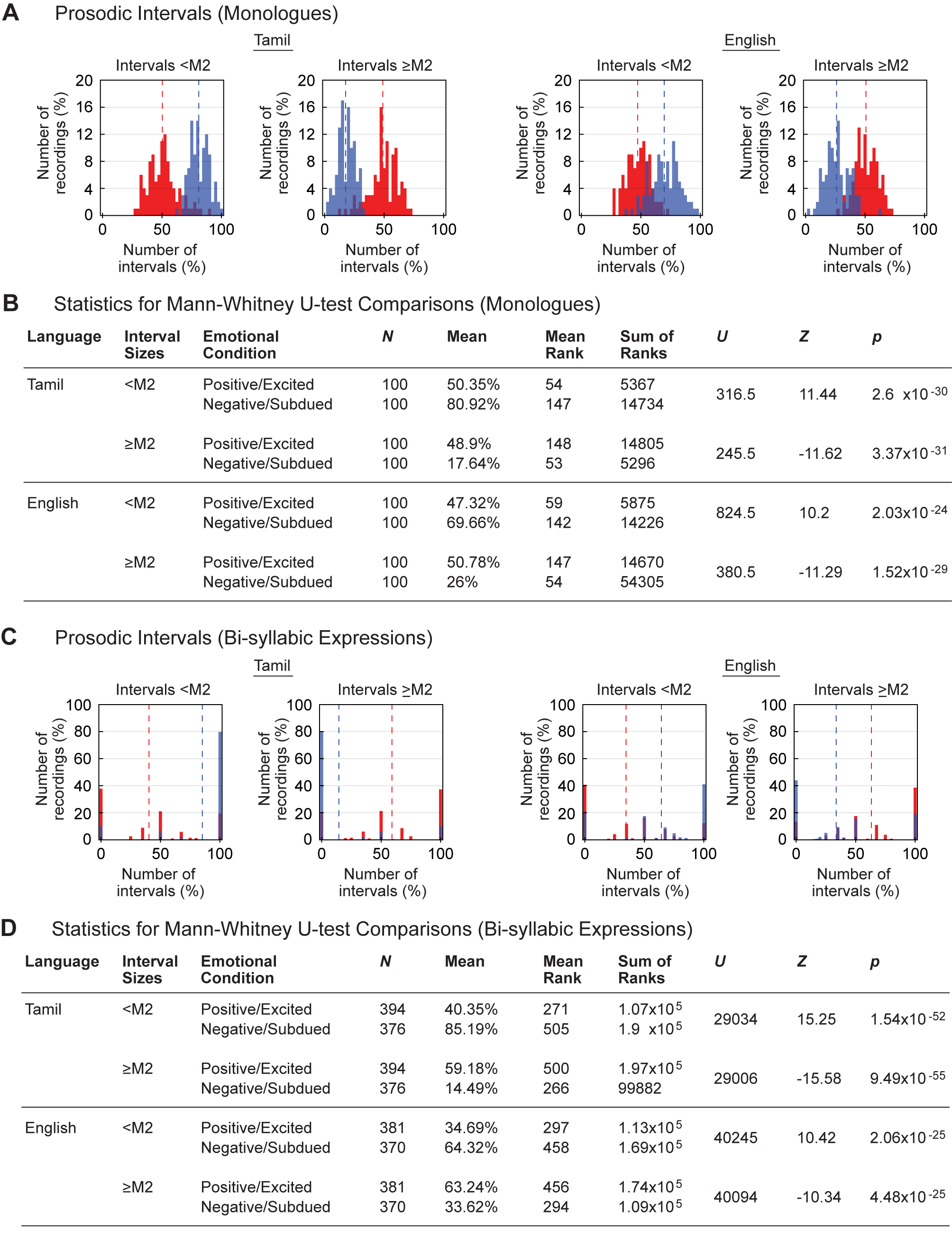

Supplement: Figure S4 — Complete statistics for prosodic interval comparisons. (A) Overlays of the distributions underlying the mean percentages shown in the insets of the monologue panels of Figure 5 (red = positive/excited, blue = negative/subdued. purple shows overlap). Each data point represents the percentage of prosodic intervals <M2 or ≥M2 in a single recording. Dashed lines indicate the means of the individual distributions. (B) The results of the two-tailed Mann-Whitney U-tests used to assess differences between the distributions in A for statistical significance. (C and D) Data presented in the same format for bi-syllabic-expressions. (TIF) [file pone.0031942.s004.tif]

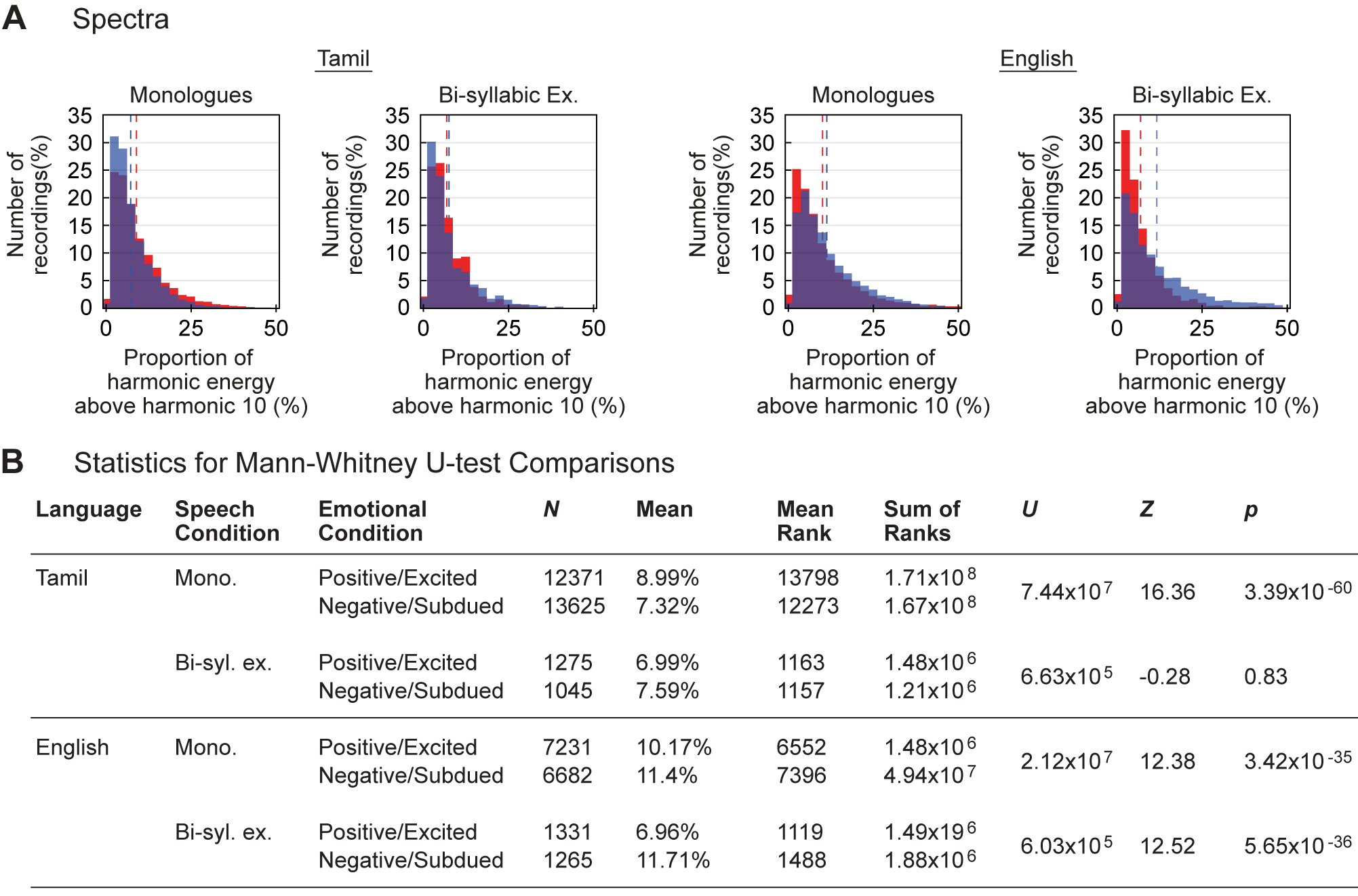

Supplement: Figure S5 — Complete statistics for average normalized spectra comparisons. (A) Overlays of the distributions underlying the mean percentages shown in the insets of Figure 6 (red = positive/excited, blue = negative/subdued. purple shows overlap). Each data point represents the average power at peaks above the tenth harmonic as a percentage of the average total power over the first 30 harmonics peaks in the voiced segments from a single recording. Dashed lines indicate the means of the individual distributions. (B) The results of the two-tailed Mann-Whitney U-tests used to assess differences between the distributions in A for statistical significance. (TIF) [file pone.0031942.s005.tif]
