# Supplementary material for: Expression of Emotion in Eastern and Western Music Mirrors Vocalization
Source: PLoS One. 2012 Mar 14;7(3):e31942. doi: 10.1371/journal.pone.0031942 (PMC3303771; doi:10.1371/journal.pone.0031942)
Supplement: Table S1 — Comparison of fundamental and formant frequencies in Tamil and English speech. (A) Comparison of mean fundamental frequencies and mean peak frequencies for the first and second formants in Tamil and English speech (from the monologue recordings). The results of two-tailed independent samples t-tests used to assess language differences for statistical significance are also shown. (B) Same as (A), but for the bi-syllabic expression recordings. Praat's [20] “To Formant” linear predictive coding algorithm was used with default settings (‘number of formants’ = 5, ‘maximum formant’ for male/female = 5 kHz/5.5 kHz) to calculate the formant frequencies. (DOC) [file pone.0031942.s006.doc]

**Table S1. Statistics for melodic interval comparisons.**

| **Musical**  **Genre** | **Interval**  **Sizes** | **Positive/excited**  ***Raga* or Major Melodies** | | | **Negative/Subdued**  ***Raga* of Minor Melodies** | | | **Statistics** | | |
| --- | --- | --- | --- | --- | --- | --- | --- | --- | --- | --- |
|  |  | *M* | *SD* | *N* | *M* | *SD* | *N* | *d.f.* | *t* | *p* |
| Carnatic | <200  cents | 19.84% | 8.68% | 100 | 59.84% | 17.99% | 100 | 192 | -19.45 | 2.9x10^-47^ |
|  | ≥200  cents | 80.11% | 8.73% | 100 | 40.16% | 17.99% | 100 | 192 | 19.41 | 3.8x10^-47^ |
| Western  Classical | <200  cents | 32.07% | 17.15% | 100 | 38.87% | 17.6% | 100 | 940 | -5.9 | 1.6x10^-8^ |
|  | ≥200  cents | 66.89% | 17.37% | 100 | 60.26% | 17.64% | 100 | 940 | 5.7 | 3.9x10^-43^ |
