# Supplementary material for: Expression of Emotion in Eastern and Western Music Mirrors Vocalization
Source: PLoS One. 2012 Mar 14;7(3):e31942. doi: 10.1371/journal.pone.0031942 (PMC3303771; doi:10.1371/journal.pone.0031942)
Supplement: Text S1 — Discussion of fundamental and formant frequencies in English and Tamil speech. This file discusses the results of the comparison of fundamental and formant frequencies in Tamil and English shown in Table S1. (DOC) [file pone.0031942.s007.doc]

**Text S1 Discussion of fundamental and formant frequencies in English and Tamil speech.**

Although there was no significant difference in the average fundamental frequency of the English and Tamil speakers, significant differences were observed in the average peak frequencies of the first and second formants (Table S1). In general, the first formant in Tamil speech was lower than in English speech, and the second formant was higher. Assuming that the vocal tracts of the American and Indian speakers are not different anatomically, this comparison of frequency characteristics indicates that the differences in average spectra between emotional conditions in Tamil and English arise from differences in the distributions of vowel phones in these languages.
