# Supplementary material for: Expression of Emotion in Eastern and Western Music Mirrors Vocalization
Source: PLoS One. 2012 Mar 14;7(3):e31942. doi: 10.1371/journal.pone.0031942 (PMC3303771; doi:10.1371/journal.pone.0031942)
Supplement: Text S2 — Further discussion of spectral similarities This file compares past [19] and present analyses of speech spectra and considers questions of perceptual relevance. The potential importance of speech spectra to the affective impact of harmonic rather than melodic arrangements of musical tones is also discussed. (DOC) [file pone.0031942.s008.doc]

**Text S2 Further discussion of spectral similarities**

*Past and Present Analyses of Speech Spectra*. Previous work from our lab [1] compared the spectra of negative/subdued and positive/excited speech to the tonality of the Western melodies composed in the major and minor modes. Similarities were found with respect to (1) the fundamental frequencies of speech and the implied fundamentals of major and minor intervals, and (2) the harmonic ratios emphasized by the first and second speech formants and the defining ratios of major and minor intervals. The present spectral analysis is similar to the earlier formant ratio analysis in that both examine spectral energy as a function of harmonic number. The present analysis, however, is more general in that it assesses overall harmonic energy rather than focusing on ratios of particular harmonics. This is an improvement in the sense that no assumptions are made about the perceptual importance of particular harmonics. Nevertheless, the results of the present analysis are consistent with those of the formant ratio analysis in ref. [19]. In both cases, there is a pattern of relatively more energy at higher numbered harmonics in negative/subdued compared to positive/excited speech. With respect to the harmonic ratios emphasized by the first and second formants, this pattern results in the increased prevalence of minor intervals (defined by ratios of higher numbers) in negative/subdued compared to positive/excited speech.

*Perceptual Relevance?* Given the similarities between emotional speech spectra and major/minor intervals, the question remains whether listeners appreciate these similarities. While implied fundamentals can readily be heard for chords of harmonically related tones [2,3], they are not typically perceived in harmonically disparate chords (such as major or minor seventh dyads), or in broken chords where the notes are sounded sequentially with a time interval greater than ~45 ms [4]. With respect to musical intervals between speech harmonics, an argument against their perceptual relevance is that speech sounds are heard as integrated wholes rather than collections of individual harmonics. Nevertheless, experiments with equal amplitude harmonic complex tones (with fundamental frequencies in the range of speech) have shown that individual harmonics up to at least the fifth or eighth can be “heard out” [5], implying that the relations between them may also be perceivable (further experiments with more naturalistic sounds are required to determine if specific harmonics beyond the eighth can be appreciated in speech). Another point worth noting is that stimuli (or aspects of stimuli) need not rise to the level of conscious perception to affect the nervous system or the associations it generates.

*Melody vs. Harmony.* Whereas speech prosody is directly comparable to the arrangement of musical tones over time (melody), its relationship to simultaneous arrangements of musical tones (harmony) is uncertain. Speech spectra, on the other hand, are similar to chords in that both entail the simultaneous sounding of multiple frequencies. Thus, spectral similarities between emotional speech and musical tonality may be more effectively considered from this perspective.

**References**

1. Bowling DL, Gill K, Choi J, Prinz J, Purves D (2010) Major and minor music compared to excited and subdued speech. Journal of the Acoustical Society of America 127: 491-503.
2. Helmholtz H (1885) Lehre von den tonempfindungen (On the sensations of Tone). 4^th^ Edition, translated by Ellis AJ. Dover. New York, NY. pp. 152-159.
3. Terhardt E, Stoll G, Seewann M (1982) Algorithm for extraction of pitch and pitch salience from complex tonal signals. Journal of the Acoustical Society of America 71: 679-688.
4. Grose JH, Hall III JW, Buss E (2002) Virtual pitch integration for asynchronous harmonics. Journal of the Acoustical Society of America 112: 2956-2961.
5. Plomp R (1964) The ear as a frequency analyzer. The Journal of the Acoustical Society of America 36:1628-1636.
